# Automatic segmentation of prostate zonal anatomy on MRI: a systematic review of the literature

**DOI:** 10.1186/s13244-022-01340-2

**Published:** 2022-12-21

**Authors:** Carine Wu, Sarah Montagne, Dimitri Hamzaoui, Nicholas Ayache, Hervé Delingette, Raphaële Renard-Penna

**Affiliations:** 1 Sorbonne Université, Paris, France; 2 Academic Department of Radiology, Hôpital Tenon, Assistance Publique des Hôpitaux de Paris, 4 Rue de La Chine, 75020 Paris, France; 3 Academic Department of Radiology, Hôpital Pitié-Salpétrière, Assistance Publique des Hôpitaux de Paris, Paris, France; 4 GRC N° 5, Oncotype-Uro, Sorbonne Université, Paris, France; 5 Inria, Epione Team, Sophia Antipolis, Université Côte d’Azur, Nice, France

**Keywords:** Artificial intelligence, Deep learning, Magnetic resonance imaging, Prostate cancer

## Abstract

**Objectives:**

Accurate zonal segmentation of prostate boundaries on MRI is a critical prerequisite for automated prostate cancer detection based on PI-RADS. Many articles have been published describing deep learning methods offering great promise for fast and accurate segmentation of prostate zonal anatomy. The objective of this review was to provide a detailed analysis and comparison of applicability and efficiency of the published methods for automatic segmentation of prostate zonal anatomy by systematically reviewing the current literature.

**Methods:**

A Preferred Reporting Items for Systematic Reviews and Meta-Analysis (PRISMA) was conducted until June 30, 2021, using PubMed, ScienceDirect, Web of Science and EMBase databases. Risk of bias and applicability based on Quality Assessment of Diagnostic Accuracy Studies 2 (QUADAS-2) criteria adjusted with Checklist for Artificial Intelligence in Medical Imaging (CLAIM) were assessed.

**Results:**

A total of 458 articles were identified, and 33 were included and reviewed. Only 2 articles had a low risk of bias for all four QUADAS-2 domains. In the remaining, insufficient details about database constitution and segmentation protocol provided sources of bias (inclusion criteria, MRI acquisition, ground truth). Eighteen different types of terminology for prostate zone segmentation were found, while 4 anatomic zones are described on MRI. Only 2 authors used a blinded reading, and 4 assessed inter-observer variability.

**Conclusions:**

Our review identified numerous methodological flaws and underlined biases precluding us from performing quantitative analysis for this review. This implies low robustness and low applicability in clinical practice of the evaluated methods. Actually, there is not yet consensus on quality criteria for database constitution and zonal segmentation methodology.

**Supplementary Information:**

The online version contains supplementary material available at 10.1186/s13244-022-01340-2.

## Key points

- Several limitations exist with current methods of automatic prostate segmentation.
- There is wide variability of databases, imaging tasks and assessment criteria.
- No preferred methodology for prostate zonal segmentation methodology and terminology was used.
- Vast majority of papers share common methodological flaws discussed in this review.

**Supplementary Information:**

The online version contains supplementary material available at 10.1186/s13244-022-01340-2.

## Introduction

Magnetic resonance imaging (MRI) is the first imaging choice for detecting and localizing prostate cancer [1, 2], based on the Prostate Imaging Reporting and Data System (PI-RADS) scoring system [3] and depending on zonal anatomy. Zonal segmentation of the prostate plays a crucial role for prostate cancer detection as the PI-RADS score differs depending on the areas studied, based on diffusion-weighted imaging (DWI) for peripheral zone lesions and T2-weighted (T2W) imaging for transitional zone lesions, but also for multiple clinical application such as reproducible prostate volume and Prostate Specific Antigen (PSA) density evaluation [4], MRI-ultrasound fusion biopsy, radiotherapy, or focal planning.

Zonal segmentation of the prostate is usually performed manually on T2W images by contouring the prostate in a slice-by-slice manner. It is extremely time-consuming, tedious, and prone to inter and intra-observer variability due to the subjective human interpretation of organ boundaries and large variability in prostate anatomy and gland intensity heterogeneity across patients [5]. There is a real need to develop automatic methods to accelerate the whole process and offer robust and accurate prostate segmentation.

Automatic zonal segmentation of the prostate is a challenging task for multiple reasons. Prostate gland is subject to large morphological variation, intra-prostatic heterogeneity, and poor contrast with adjacent tissues, making delineation of prostatic zonal contours laborious. Multi-institutional applicability can be difficult to evaluate as there is a wide technically induced variability in the image acquisition, as MRI signal intensity is not standardized and image characteristics are strongly influenced by acquisition protocol, field strength, scanner type, coil type, etc. [6].

Finally, the performances of an automated segmentation method depend in part on the database (heterogeneity of the data used, knowledge of possible selection biases), quality of ground truth (manual delineation of the prostate performed by human experts), training time and hardware requirements. First commonly used methods were based on machine learning methods, such as atlas-based registration models in which several reference images with corresponding labels are registered and deformed onto the target image [7, 8] or C-means clustering models [9, 10]. Most common methods described after 2017 are based on deep learning with convolutional neural networks (CNN) allowing automatic extraction of features and semantic image segmentation. Common architectures such as U-net [11], V-net or ResNet have been extensively used. Modification and fine tuning of existing models, by either combining multiple U-nets [12–14], adding attention modules such as squeeze and excitation [15], feature pyramid attention [16], adding blocks [17], transition layers or up-sampling strategies [18], allowed either improving accuracy of classical CNN or obtaining same accuracy with reduced memory and storage requirements.

The primary objective of this review was to provide a detailed analysis and comparison of applicability and efficiency of the published methods for automatic segmentation of prostate zonal anatomy by systematically outlining, analyzing, and categorizing the relevant publications in the field to date. We also aimed to identify methodological flaws and biases to demonstrate the need for a consensus on quality criteria for database constitution and prostate zonal segmentation methodology.

## Materials and methods

This systematic review was conducted and reported in accordance with the Preferred Reporting Items for Systematic Reviews and Meta-Analyses statement (PRISMA) [19]. The methods for performing this systematic review were registered on PROSPERO [20] database (registration number CD42021265371), and were agreed by all authors before the start of the review process to avoid bias. This study was exempt from ethical approval at our institution because the analysis involved only de-identified data.

### Data sources and search

Medical literature published in the English language published until 30 June 2021 was searched in multiple databases (Medline, Science direct, Embase and Web of Science) using the following terms:

(prostatic OR prostate) AND (automated OR automatic) AND (segmentation OR segmented) AND (zone OR zonal) AND (\"magnetic resonance\" OR mri OR \"magnetic resonance\"OR mri OR mr) AND (\"artificial intelligence\" OR \"deep learning\" OR \"machine learning\") and all possible combinations.

No beginning date was applied.

### Study selection

**Fig. 1:**
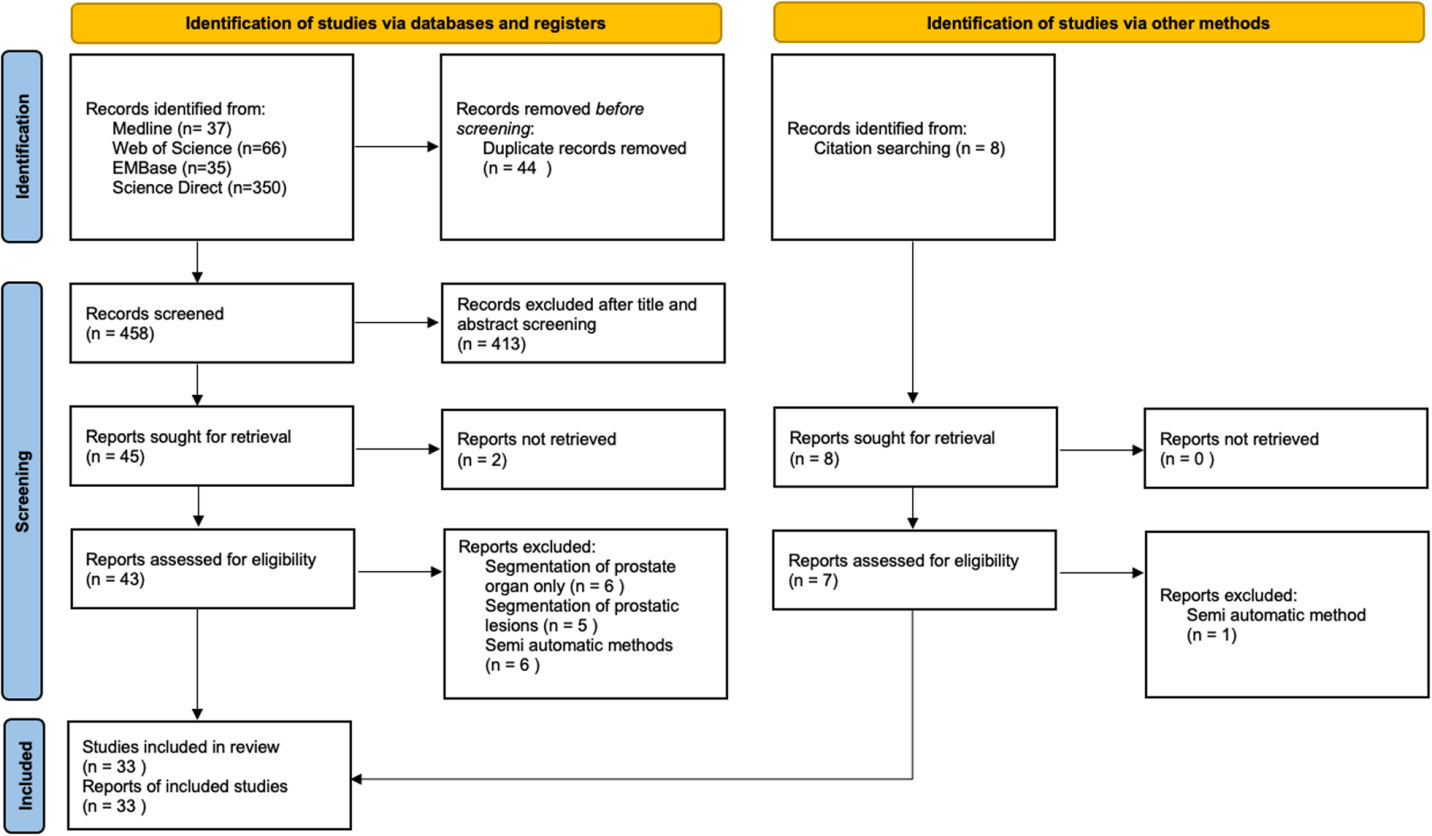
Flow diagram based on Preferred Reporting Items for Systematic Reviews and Meta-Analyses (PRISMA) recommendations for systematic reviews

Full-text selection was independently performed by two radiologists, one experimented radiologist specialized in uroradiology and prostate imaging (S.M., 5 years in prostate imaging, with more than 1000 cases of prostate MRI per year) and one radiology fellow specialized in uroradiology and prostate imaging (C.W., 1 year in prostate imaging, with more than 1000 cases of prostate MRI per year). A third experimented professor of radiology specialized in prostate imaging (R.R.P., 15 years in prostate imaging, with more than 1000 cases of prostate MRI per year) intervened in case of disagreement. We summarized search strategy details for each database in Fig. 1.

We imported all articles retrieved into the reference manager Zotero and removed all duplicates. The same two radiologists (C.W., S.M.) then independently and manually screened titles and abstracts of the resultant database to ensure relevance. Articles that were obviously out of the scope of the research topic were excluded at this stage. Subsequently, all the remaining articles full texts were retrieved and read, applying inclusion and exclusion criteria (explained below) with conflicts resolved by consensus with the third reviewer. Reference lists of these relevant articles were also reviewed for possible papers missed in the primary search, and those papers were screened using the same initial inclusion and exclusion criteria.

### Selection criteria

#### Inclusion criteria

Articles were included if they were original articles, used machine learning or deep learning algorithms and aimed to segment prostate human MRI images by zonal anatomy, using a fully automated method with manual segmentation as ground truth.

#### Exclusion criteria

Articles were excluded if they were commentaries, editorials, letters, case reports or abstracts. Were also excluded articles with semi-automated segmentation methods, no description of segmentation method, segmentation of the whole gland (WG), or prostate cancer without zonal anatomy, absence of similarity metrics or of evaluation against ground truth segmentations.

### Data collection and extraction process

The qualifying papers were then reviewed, and various data of the studies were extracted and tabulated prior to analysis (Table 1).

**Table 1:** Data extraction

Sources	Patients	Data	Flow and timing	Reference standard	Test
Scientific database	Public or in-house database	Vendor	Cross-validation	Type of annotation	Validation or test on external data
Title	Eligibility criteria: inclusion and exclusion criteria	Field	Splitting in training, validation and test set	Annotation tool *if used*	Performance metrics
Authors	Sample size	Array		Number of annotators	Results based on DSC
Year of publication	Ethic consent	Field of view		Ground truth segmentation and rationale	
Journal name	Presence of benign prostate hypertrophia	Pre-processing		Measurements of inter- and intra-rater variability *if any*	
	Presence of prostate cancer	Post-processing		Type of annotators	
	Percentage of prostate cancer	Number of vendors		Experience of annotators	
	Uni or multicentric	Slice thickness			
	Prospective or retrospective	Type of slice and sequence			
		Cross-validation			

*DSC* dice similarity coefficient

#### Assessment of methodological quality

The two same radiologists (C.W., S.M.) independently assessed and extracted data from each of the included articles, using the Quality Assessment of Diagnostic Accuracy Studies tool-2 (QUADAS-2) framework [21] adjusted with topics from the Checklist for Artificial Intelligence in Medical Imaging (CLAIM) [22] to evaluate the risk of bias and applicability for each selected study, with conflicts resolved by consensus with the third reviewer.

Extracted data were tabulated, synthesized, and evaluated for methodological flaws and applicability of the proposed techniques.

## Results

**Fig. 2:**
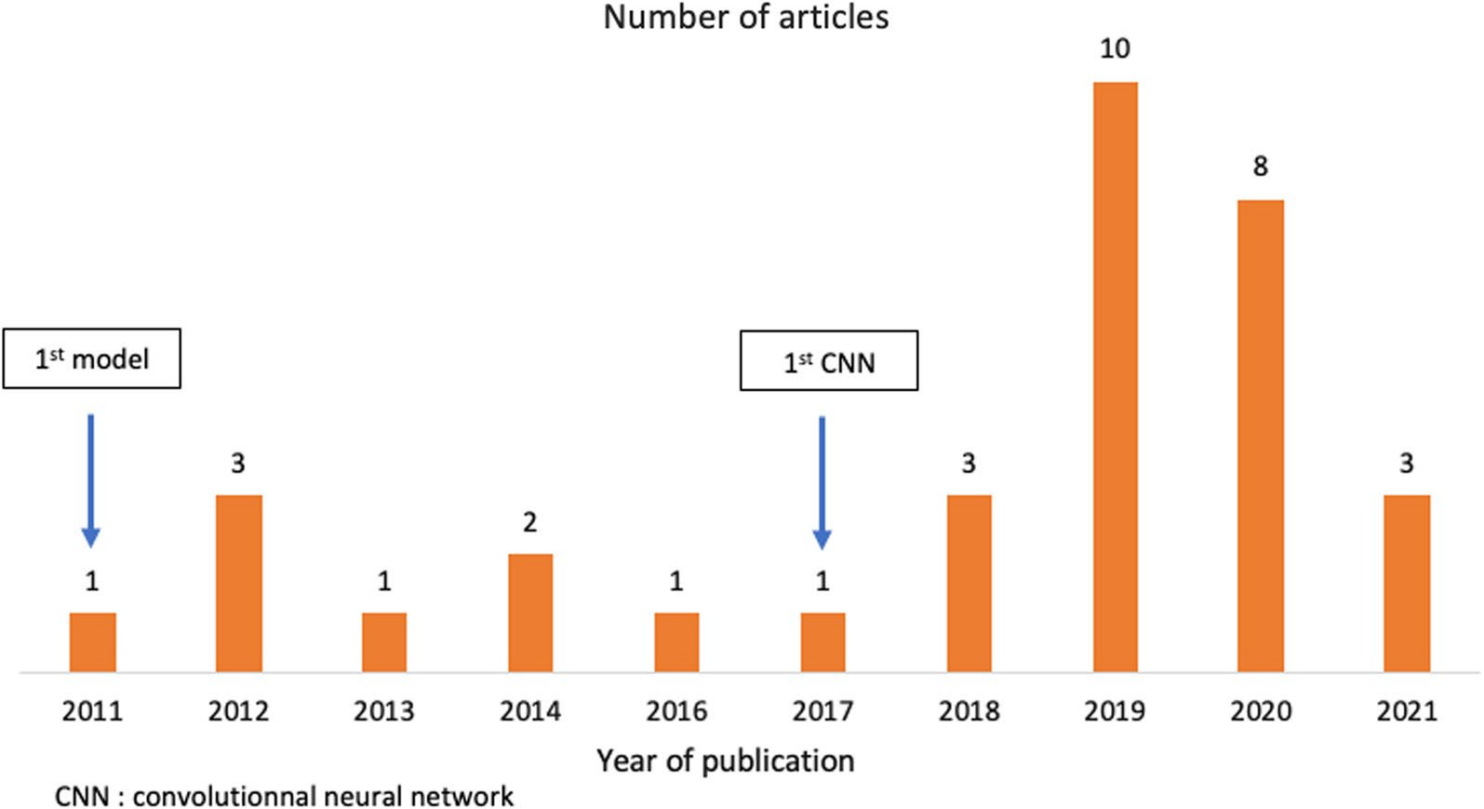
Chronological distribution of the 33 reviewed articles. 1st model for prostate zonal anatomy segmentation was published in 2011. 1st convolutional neural network (CNN) was published in 2017

After removing duplicates, 458 articles were remaining. Final consensus was reached yielding a total of 33 articles [6–10, 12–18, 23–43] (Figs. 1, 2).

### Datasets

#### Training, validation, and test sets

All articles used retrospective datasets.

Wide heterogeneity in training, validation and test datasets was found (Table 2).

**Table 2:** Overview of types of databases used with training, validation and test sets distribution

First author, year of publication	Inclusion criteria	Presence of PCa	Number of patients (total)	Training			Validation		Test		
				Total	Public data	In-house data	Cross-validation	Validation data	Total	Public data	In-house data
Cuocolo et al. [43]	✓	✓	204	79	79^(A)^	0	Fivefold	20	105	105^(A)^	0
Bardis et al. [42]	✓	✓	242	146	0	145	0	48	48	0	48
Lai et al. [41]	✓	✓	115	80	80^(A)^	0	Fivefold	20	15	15^(A)^	0
Nai et al. [18]	✓	✓	160	120	120^(A)^	0	0	20	20	20^(A)^	0
Sanford et al. [40]	✓	✓	1054	518 + 162^§^	0	680	0	130 + 42^§^	202	0	202
Aldoj et al. [39]	✓	✓	188	106	106^(A)^	0	Fourfold	35	47	20^(A)^	0
Zavala-Romero et al. [6]	✓	✓	550	198 or 297	297^(A)^	198	0	0	variable	33^(A)^	22
Lee et al. [38]	✓	✓	330	260 *(for WG)* or 162 *(for TZ)*	0	260 *(for WG)* or 162 *(for TZ)*	0	0	70 *(for WG)* or 50 *(for TZ)*	0	50
Liu et al. [37]	✓	✓	351	218	218^(A)^	0	0	45	92	45^(A)^	47
Qin et al. [36]	×	✓	240	162 + 45	45 ^(B)^	162	0	0	33	15 ^(B)^	18
Motamed et al. [35]	×	Unknown	681	291 *(source)* + variable *(target)*	0	406	0	97	145 *(source)* + 33 *(target)*	0	178
Zabihollahy et al. [13]	✓	✓	225	80	0	80	0	20	125	0	125
Padgett et al. [8]	✓	✓	61	Variable	0	Variable	0	0	Variable	0	1
Rundo et al. [15]^1^	×	✓	80	Variable	Variable^(B)^	Variable	0	0	Variable	Variable^(B)^	Variable
Meyer et al. [34]	✓	✓	98	58	58^(A)^	0	Fourfold	20	20	20^(A)^	0
Liu et al. [16]	✓	✓	359	200	200^(A)^	0	Fivefold	50	110	63^(A)^	46
Rundo et al. [33]^2^	×	✓	40 ^†^	Variable ^†^	^†(C)^	Variable	0	0	Variable	0	Variable
Hambarde et al. [32]	×	Unknown	52	42	0	42	0	0	10	0	10
Jensen et al. [31]	✓	✓	40	32	0	32	Fivefold	2	8	0	8
Khan et al. [17]	×	✓	80	35	35^(B)^	0	0	15	30	30^(B)^	0
Cheng et al. [30]	×*	✓	225	116 +/−^†^	8^(A)^	108	0	0	Variable	Variable^(A+C)^	27
Zhu et al. [12]	✓	✓	163	76	0	76	0	36	51	0	51
Mooij et al. [29]	0	Unknown	53	36	0	36	Fivefold	9	8	0	8
Can et al. [28]	0	Unknown	29	12	12^(B)^	0	0	7	10	10^(B)^	0
Clark et al. [14]	×*	✓	154	115	78^(C)^	37	0	0	38	12^(C)^	26
Chilali et al. [9]	✓	✓	55	30	30 *(Prostatlas)*	0	0	0	25	13^(C)^	12
Makni et al. [10]	✓	✓	31	? *(simulated images)*	0	0	0	0	31	0	31
Chi et al. [27]	✓	Unknown	8	4	0	4	0	0	4	0	4
Toth et al. [26]	✓	✓	40	Variable	0	Variable	0	0	Variable	0	Variable
Litjens et al. [7]	×	Unknown	48	48	0	47	0	0	1	0	1
Moschidis and Graham [25]	✓	×	22	Variable	0	Variable	0	0	Variable	0	Variable
Yin et al. [24]	×	✓	522 *(images)*	261 *(images)*	0	261 *(images)*	Fivefold	52 *(images)*	261 *(images)*	0	261 *(images)*
Makni et al. [23]	✓	✓	31	?	0	0	0	0	31	0	31

*Not specified for in-house data

^†^+/− 50 patients from PROMISE12 dataset used for pre-training of WG segmentation

^§^Pre-training data + data for transfer learning

^(A)^Public data used is PROSTATE-X

^(B)^Public data used is NCI-ISBI

^(C)^Public data used is PROMISE12

^1^Rundo et al., USE-Net: incorporating Squeeze-and-Excitation blocks into U-Net for prostate zonal segmentation

of multi-institutional MRI datasets [30]

^2^Rundo et al., CNN-based Prostate Zonal Segmentation on T2-weighted MR Images: A Cross-dataset Study[27]

*PCa* prostate cancer, *WG* whole gland, *TZ* transition zone

Performance testing of the algorithms can be done on same source than for the development or use different source of data, and based on either public data, private data or a combination of both. Public data were used in 15/33 articles for testing. Only 7 studies [6, 9, 14, 30, 33, 36, 37] used both private and public data for testing, allowing better generalizability of their algorithms. None of them used prospective data for validation and testing.

Most used public datasets were PROSTATEx [44], NCI-ISBI 2013 [45] and PROMISE12 [46] (Additional file 1: Table S1).

Eight authors applied cross-validation, using a subset of available dataset as training set, while the remaining data constituted the test set to evaluate the segmentation performance and accuracy. Nine reported using cross-validation for testing, averaging the results from the different rounds, hence adding bias.

#### Technique

We identified major technical differences in datasets regarding the number of vendors, field strength, type of coils, sequences, slice thickness, field of view (FOV) and input data used for automatic segmentation (Table 3). Less than half (14/33) studies used more than one type of vendors and 7/33 used both 1.5T and 3T MRI machines. More than 2/3 (24/33) used mono-modal input, mainly T2-weighted planes, in combination with apparent diffusion coefficient (ADC) map in one study [13] or with multiparametric and multi-incidence MR images in another [9]. The slice thickness of T2-weighted axial planes was consistent with the PI-RADS v2.1 recommendations in 13/33 studies (≤ 3 mm), which was not the case for the public data base PROSTATEx (3.6 mm). Only 7 studies provided sequence details (type of sequence, slice thickness, FOV) used for ground truth manual segmentation.

**Table 3:** Input MRI parameters (number of vendors, type of field, type of coil, input sequences)

First author, year of publication	Vendors				Type of coil		Field strenght		Data input		
	Number of vendors	Philips	GE	Siemens	ERC	SC	1.5T	3T	Mono-parametric	Sequence	Slice thickness*
Cuocolo et al. [43]	1	–	–	✓	–	✓	–	✓	✓	Axial T2W	3.6
Bardis et al. [42]	2	✓	–	✓	–	✓	–	✓	✓	Axial T2W	3.0
Lai et al. [41]	1	–	–	✓	–	✓	–	✓	×	Axial T2W + DWI + ADC	3.6
Nai et al. [18]	1	–	–	✓	–	✓	–	✓	×	Axial T2W + DWI + ADC	3.6
Sanford et al. [40]	3	✓	✓	✓	✓	✓	✓	✓	✓	Axial T2W	3.0
Aldoj et al. [39]	1	–	–	✓	–	✓	–	✓	✓	Axial T2W	3.6
Zavala-Romero et al. [6]	2	–	✓	✓	–	✓	–	✓	✓	3 planes T2W	3.6
Lee et al. [38]	1	–	–	✓	–	✓	–	✓	✓	Axial + sagittal T2W	3.0
Liu et al. [37]	1	–	–	✓	–	✓	–	✓	✓	Axial T2W	3.0–3.6
Qin et al. [36]	at least 2	✓	?	✓	✓	✓	✓	✓	×	Axial T2W + ADC	3.6
Motamed et al. [35]	2	✓	–	✓	?	?	?	?	✓	DWI	3.0
Zabihollahy et al. [13]	1	–	✓	–	–	✓	–	✓	×	Axial T2W + ADC	3.0–4.0
Padgett et al. [8]	2	–	✓	✓	?	?	–	✓	✓	Axial T2W	2.5
Rundo et al. [15]^1^	2	✓	–	✓	–	✓	–	✓	✓	Axial T2W	1.25–4.0
Meyer et al. [34]	1	–	–	✓	–	✓	–	✓	✓	Axial T2W	3.0
Liu et al. [16]	1	–	–	✓	–	✓	–	✓	✓	Axial T2W	3.6
Rundo et al. [33]^2^	2	✓	–	✓	–	✓	–	✓	✓	Axial T2W	1.25–3.0
Hambarde et al. [32]	1	✓	–	–	?	?	✓	–	✓	Axial T2W	5.0
Jensen et al. [31]	2	–	✓	✓	✓	✓	✓	✓	✓	Axial T2W	1.5–3.0
Khan et al. [17]	2	✓	–	✓	✓	✓	✓	✓	✓	Axial T2W	3.0–4.0
Cheng et al. [30]	multiple	?	?	✓	✓	✓	?	✓	✓	Axial T2W	3.0
Zhu et al. [12]	1	✓	–	–	–	✓	–	✓	×	Axial T2W + DWI	4.0
Mooij et al. [29]	?	?	?	?	?	?	?	?	✓	3D T2W	3.6
Can et al. [28]	2	✓	–	✓	✓	✓	✓	✓	✓	Axial T2W	3.0–4.0
Clark et al. [14]	multiple	✓	?	?	✓	✓	✓	✓	✓	DWI	?
Chilali et al. [9]	3	✓	✓	✓	✓	✓	✓	✓	✓	Axial T2W	3.0–4.0
Makni et al. [10]	1	✓	–	–	?	?	✓	–	×	Axial T2W + DWI + CE	1.25
Chi et al. [27]	1	–	–	✓	–	✓	–	✓	×	Axial T2W + ADC	3.3–3.75
Toth et al. [26]	?	?	?	?	✓	–	–	✓	✓	Axial T2W	3.0
Litjens et al. [7]	?	–	–	–	?	?	?	?	×	Axial T2W + ADC	4.0
Moschidis and Graham [25]	1	✓	–	–	–	✓	✓	–	✓	3D T2W	?
Yin et al. [24]	1	✓	–	–	✓	✓	–	✓	✓	Axial T2W	3.0
Makni et al. [23]	1	✓	–	–	–	✓	✓	–	×	Axial T2W + DWI + CE	2.5

*ERC* endorectal coil, *SC* Surface coil, *T2W* T2-weighted, *DWI* diffusion-weighted imaging, *ADC* apparent diffusion coefficient, CE contrast-enhanced

*Slice thickness in mm, when axial T2W slices used

?Not reported

^1^Rundo et al., USE-Net: incorporating Squeeze-and-Excitation blocks into U-Net for prostate zonal segmentation

of multi-institutional MRI datasets [30]

^2^Rundo et al., CNN-based Prostate Zonal Segmentation on T2-weighted MR Images: A Cross-dataset Study[27]

### Zonal anatomy

**Fig. 3:**
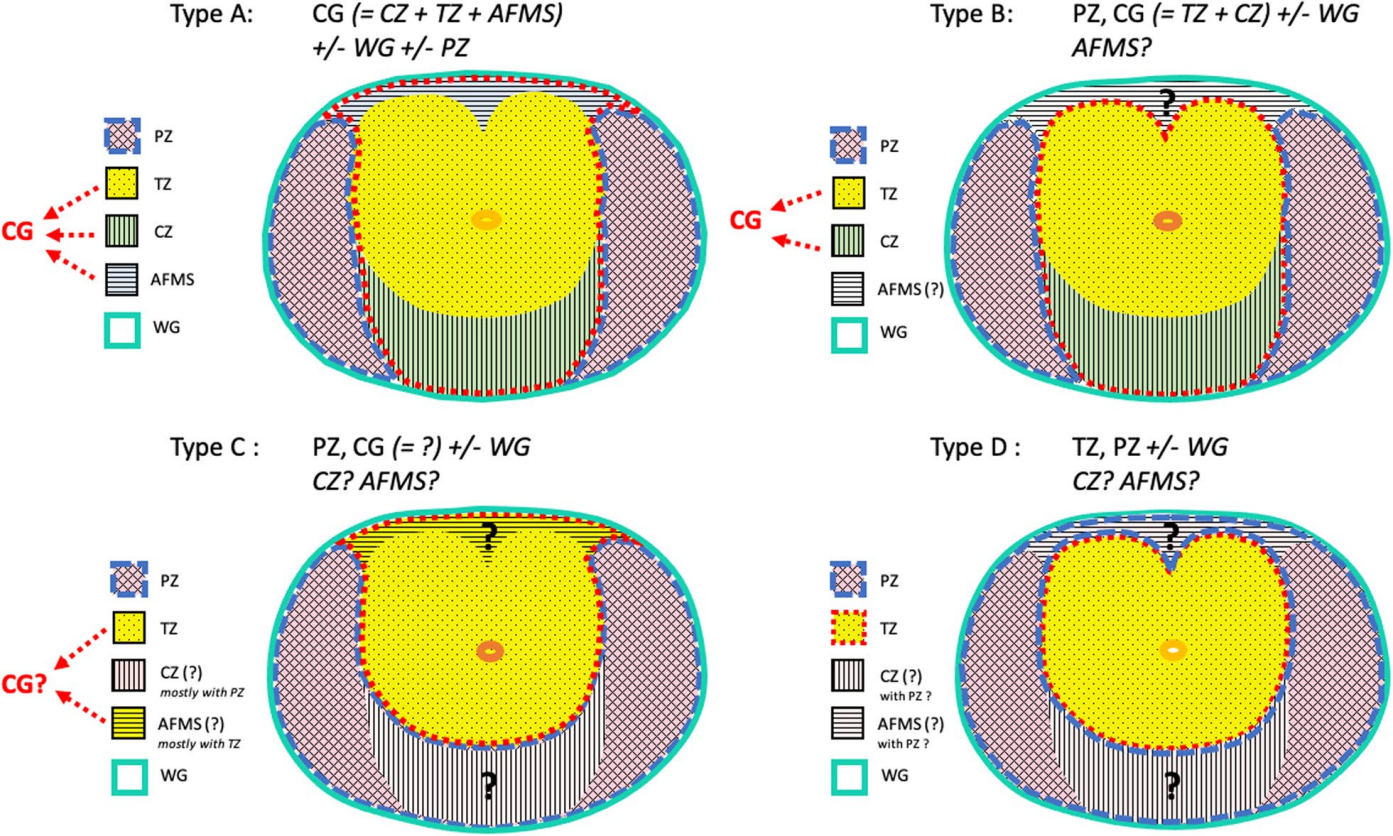
Schematic of the four major types of protocol of zonal segmentation. Type A: articles for which “central gland” included CZ, TZ and AFMS. Type B: articles for which “central gland” included TZ and CZ. No details for AFMS. Type C: articles which did not provide details for AFMS, CZ or CG. CZ seemed to be mostly segmented PZ, while AFMS seemed to be mostly segmented with TZ, usually called “CG”. Type D: articles which did not provide details for AFMS or CZ. CZ and AFMS seemed to be mostly segmented with PZ. *CZ* central zone, *TZ* transition zone, *AFMS* anterior fibro-muscular stroma, *PZ* peripheral zone, *CG* central gland

We found 18 different types of very heterogeneous and unclear terminologies of zonal anatomy (Fig. 3, Additional file 1: Fig. S1). Out the 33 articles reviewed, less than 1/4 (8/33) [23, 25, 32, 34, 36, 37, 40, 43] provided precise terminology and segmentation protocol. Frequently the inappropriate term “central gland” (CG) was used, with ambiguous definition of central zone (CZ) and anterior fibro-muscular stroma (AFMS) alternatively included in peripheral zone (PZ) or transition zone (TZ), or mainly not described at all. Two studies mis-used the term “central zone” to refer to the “central gland” [27, 39].

### Ground truth

Manual delineation of the prostate gland performed by human experts was used to generate ground truth (Table 4).

**Table 4:** Type of ground truth segmentation

First author, year of publication	Annotation		Annotators			
	Type	Tool	Qualification	Number	Type of reading	Experience*
Cuocolo et al. [43]	Software	itk-SNAP	(A)	4	Splitted^¶^	2 to 5
Bardis et al. [42]	Software	In house	(A)	12	Stratified	10
Lai et al. [41]	Manual	–	(A)	1	–	10
Nai et al. [18]	Software	MITK	Medical physicist	4	Stratified	2 to 10
Sanford et al. [40]	Software	pseg	(A)	1	–	10
Aldoj et al. [39]	Manual	–	(A)	1	–^₸^	"Expert"
Zavala-Romero et al. [6]	Manual	–	(A) + (B)	3	Stratified	10
Lee et al. [38]	Manual	–	(A)	2	?	4
Liu et al. [37]	Software	Osirix	(C) + (A)	More than 2	Stratified	10 and 19
Qin et al. [36]	Manual	–	(A)^†^	?	–	?
Motamed et al. [35]	Manual	–	(A)	2	?	4 and 6
Zabihollahy et al. [13]	Software	itk-SNAP	(A)	4	Splitted	5 and 14
Padgett et al. [8]	Manual	–	(B)	2	Blinded^§^	10 and 26
Rundo et al. [15]^1^	Manual	–	?	Multiple	?	"Expert"
Meyer et al. [34]	Software	3DSLICER	Medical student + urologist + (A)	4	Stratified	"Expert"
Liu et al. [16]	Software	Osirix	(C) + (A)	7	Stratified	10–15
Rundo et al. [33]^2^	?	?	(A)	Multiple	?	?
Hambarde et al. [32]	?	?	(A)	Multiple	?	?
Jensen et al. [31]	Software	?	(A)	1	–	"Expert"
Khan et al. [17]	Manual	–	(A)	3	?	?
Cheng et al. [30]	Software	pseg	(A)	1	–	10
Zhu et al. [12]	Manual	–	?	2	?	More than 5
Mooij et al. [29]	Manual	–	?	?	?	?
Can et al. [28]	Manual	–	(A)	3	?	?
Clark et al. [14]	Manual	–	(A)	1	?	?
Chilali et al. [9]	Manual	–	(A)	1	–	15
Makni et al. [10]	Manual	–	(A)	3	Blinded	"Expert"
Chi et al. [27]	Manual	–	(A)	1	–	5
Toth et al. [26]	Software	3DSLICER	(A)	1	–	"Expert"
Litjens et al. [7]	Manual	–	(A)	3	?	?
Moschidis and Graham [25]	Manual	–	(A)	2	?	?
Yin et al. [24]	Manual	–	"Radiologist-trained operators"	2	Splitted	?
Makni et al. [23]	Manual	–	(A)	3	Blinded	4, 6 and 9

(A) Radiologist

(B) Radiation oncologist

(C) Research fellow

? Data not reported

*Experience of reader(s), in years

^†^Unclear for PROMM, in-house data

^¶^Splitted but consensus per binome resident-senior

^₸^Only one reading for ground truth segmentation but evaluation of intra and inter observator variability on some masks

^§^Measure of inter- observator variability for 10 masks

Splitted: database is divided such as each set of images is read only once, resulting in an equivalent of single reader

Stratified: first reading (mostly by a less experienced reader) subsequently corrected by a more experience reader

Blinded: blinded reading by at least 2 readers

^1^Rundo et al., USE-Net: incorporating Squeeze-and-Excitation blocks into U-Net for prostate zonal segmentation

of multi-institutional MRI datasets [30]

^2^Rundo et al., CNN-based Prostate Zonal Segmentation on T2-weighted MR Images: A Cross-dataset Study[27]

#### Annotation tool

Twenty studies (61%) reported using manual contouring, while a third (11/33) reported using annotation tools. One team [31] specified that the radiologist did not delineate zones on all slices but relied on interpolation performed by their annotation tools. Two studies [32, 33] did not provide any information.

#### Qualifications of annotators

Most studies (27/33, 81%) reported a radiologist or a radiation oncologist as human expert. In 3 papers, no detail was provided on annotators qualification, although one [15] specified using an “expert” reader. Definition of an “expert” reader was mostly unclear with no specification of number of MRI they interpreted, for example [10, 15, 26, 31, 34, 39].

#### Number of readers

Number of readers and their experience are described in Table 4. Number of readers was not available in two studies. While 2/3 of teams (22/33) reported using more than one reader, with splitted, stratified or blinded reading approaches, 7 did not provide information on reading approach.

#### Intra and inter-rater variability

Inter-rater variability for annotations was rated in only 4 studies [7, 10, 23, 39]. Some studies used alternative techniques to approach better homogeneity of ground truth. In [13], the four radiologists met for a training session and together segmented two example patients to achieve a similar methodology for the rest of the dataset, using only experienced radiologists. In [6], the contours segmented by three radiologists were cross-checked and reviewed by two radiation oncologists, resulting in better homogeneity of ground truth. In [18], the initial prostate masks were drawn by two students who were trained in segmenting prostate zones.

### Risk of bias and quality assessment

**Fig. 4:**
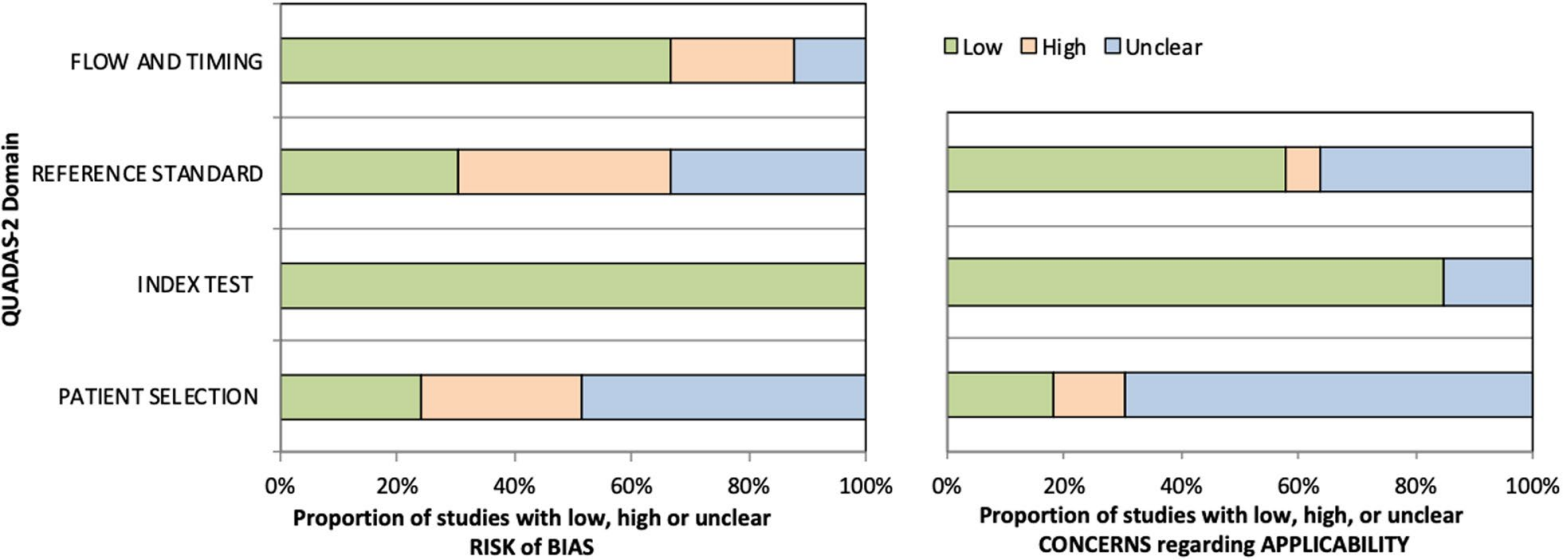
Stacked bar charts showing results of quality assessment for risk of bias and applicability of included studies. QUADAS-2 scores for methodologic study quality are expressed as the percentage of studies that met each criterion. For each quality domain, the proportion of included studies that were determined to have low, high, or unclear risk of bias and/or concerns regarding applicability is displayed in green, orange, and blue, respectively. QUADAS-2: Quality Assessment of Diagnostic Accuracy Studies 2

The detailed results are presented in Fig. 4 and Additional file 1: Table S2.

Regarding patient selection, we considered a low risk of bias if there were clear data inclusion and exclusion criteria, inclusion of patients with and without PCa. Models were considered less applicable if datasets were composed of only one type of scanners or if no information was specified.

For reference standard, number of readers and type of reading for ground truth segmentation were reviewed.

Clear partitioning of the database (into training, validation, and test sets) was needed to waive risk of bias for flow and timing. Some articles used cross-validation methods without keeping a clear independent test dataset [6–8, 15, 25, 26, 30, 33, 36].

Overall, all 33 included studies were judged to have a low risk of bias in the domain “index test” and 22 of 33 (67%) of the studies were judged to have a low risk of bias considering “flow and timing”. However, only 1/4 of the studies (8/33) were judged to have a low risk of bias in the domain “patient selection”, 1/3 (10/33) in the domain “reference standard”. Only 2 articles were judged to have a low risk of bias in all four domains.

### AI methodology

Before 2017, authors mostly used machine learning-based methods for automatic segmentation of prostatic zones. After 2017, almost all publications were based on deep learning with convolutional neural networks (CNN) (72%, 24/33). Common architectures such as U-net [11] have been extensively used, with modification and fine tuning of existing models, allowing either improved accuracy of classical networks or reduced memory and storage requirements.

Dice coefficient (DSC) and Hausdorff distance [47] were commonly used metrics. Almost all authors found inferior results for PZ than WG, CG or TZ segmentation, attributing this to the more complex shape and structure of PZ, especially within the anterior bundles. Eleven authors subsequently stratified their DSC results based on prostate height, with various methods:in three equal parts [13], in 25% apex, 50% mid gland and 25% base [39] in 30%, 40% and 30%, respectively [31]. Five authors did not provide any details on how they divided the volume.

These results as well as the remaining metrics are summarized in Table 5.

**Table 5:** Overview of segmentation methods with performance based on DSC. Number of articles reporting stratification by gland height, and reporting pre- or post-processing steps

First author, year of publication	Type	DSC results^†^				Stratification by gland height	Pre-processing details	Post-processing details
		WG	TZ	PZ	CG			
Cuocolo et al. [43]	CNN	0.9063*	–	0.7142*	0.8692*	×	✓	×
Bardis et al. [42]	CNN	0.94	0.91	0.774	–	×	✓	×
Lai et al. [41]	CNN	–	0.93	0.7004	–	×	✓	×
Nai et al. [18]	CNN	0.89*	–	0.712*	0.856*	✓	✓	×
Sanford et al. [40]	CNN	0.915	0.89	–	–	×	✓	×
Aldoj et al. [39]	CNN	0.921*	–	0.781*	0.895*	✓	✓	×
Zavala-Romero et al. [6]	CNN	0.825^a^ 0.892^b^	–	0.788 ^a^ 0.811 ^b^	–	×	✓	✓
Lee et al. [38]	CNN	0.8712	0.7648	–	–	×	✓	×
Liu et al. [37]	CNN	–	0.89*^c^ 0.87*^d^	0.80*^c^ 0.79*^d^	–	✓	✓	×
Qin et al. [36]	CNN	–	–	0.806	0.901	×	✓	✓
Motamed et al. [35]	CNN	0.89^e^ 0.85^f^	0.86^e^ 0.84^f^	–	–	×	×	✓
Zabihollahy et al. [13]	CNN	0.9533^g^ 0.9209^h^	–	0.8678^g^ 0.861^h^	0.9375^g^ 0.8989^h^	✓	✓	✓
Padgett et al. [8]	Atlas	0.83*	0.75*	0.59*	–	✓	×	×
Rundo et al. [15]^1^	CNN	–	–	0.919^i^ 0.831^j^ 0.801^k^	0.871^i^ 0.886^j^ 0.937^k^	×	✓	✓
Meyer et al. [34]	CNN	–	0.876	0.798	–	×	✓	✓
Liu et al. [16]	CNN	–	0.86^c^ 0.79^d^	0.74^c^ 0.74^d^	–	✓	✓	×
Rundo et al. [33]^2^	CNN	–	–	0.91* (with pre-training)	0.85* (with pre-training)	×	✓	✓
Hambarde et al. [32]	CNN	–	–	0.8733	–	×	✓	×
Jensen et al. [31]	CNN	–	–	0.692	0.794	✓	✓	✓
Khan et al. [17]	CNN	–	–	0.703*	0.88*	×	×	×
Cheng et al. [30]	CNN	0.9235*	–	–	0.9006*	✓	✓	✓
Zhu et al. [12]	CNN	0.927	–	0.793	–	✓	✓	×
Mooij et al. [29]	CNN	–	0.85*	0.6*	–	×	✓	×
Can et al. [28]	CNN	–	–	0.722*	0.89*	×	×	×
Clark et al. [14]	CNN	0.886^c^ 0.862^d^	0.847^c^	–	–	×	✓	×
Chilali et al. [9]	C means + Atlas	0.9478	0.7023	0.62	–	×	✓	×
Makni et al. [10]	C means	–	0.88	0.78	–	×	✓	×
Chi et al. [27]	Gaussian model	0.8	–	0.53	0.83	×	×	×
Toth et al. [26]	Active appearance model	0.81	–	0.68^l^ 0.60^m^	0.79^l^ 0.72^m^	✓	✓	×
Litjens et al. [7]	Atlas	–	–	0.75	0.8	×	×	×
Moschidis and Graham [25]	Random Forrest + Graph Cuts	–	–	–	–	×	✓	×
Yin et al. [24]	Graph Cuts	–	–	–	0.81	×	×	×
Makni et al. [23]	C means	–	–	0.76^l^	0.87^l^	✓^¶^	×	×

*CNN* convolutional neural network

^†^Dice similarity coefficient (DSC) for whole gland (WG), transition zone (TZ), peripheral zone (PZ) or central gland (CG) (*means*)

*Best results if several models were tested

^¶^no Dice Similary Coefficien (DSC) provided

^a,b^Trained on combined datasets and, respectively, tested on GE^a^ or Siemens^b^ dataset

^c,d^Respectively for testing on internal^c^ or external^d^ data

^e,f^Respectively for source^e^ or target^f^ with 115 patients for training (best results)

^g,h^Respectively for T2-weighted^g^ and apparent diffusion coefficient (ADC) map^h^

^I,j,k^Trained on combined datasets and, respectively, tested on dataset #1^i^, #2^j^ or #3^k^

^l,m^Using pre-segmented whole gland (WG)^l^, or with whole process^m^

^1^Rundo et al., USE-Net: incorporating Squeeze-and-Excitation blocks into U-Net for prostate zonal segmentation of multi-institutional MRI datasets[30]

^2^Rundo et al., CNN-based Prostate Zonal Segmentation on T2-weighted MR Images: A Cross-dataset Study[27]

## Discussion

Our systematic review highlights the high prevalence of deficiencies in methodology in the literature on automatic segmentation of prostate gland on MRI.

Since 2011, 33 studies proposed new or fine-tuned existing approaches for automatic prostatic zonal segmentation. Many studies are hampered by issues with limitation of the dataset used in the model, methodological mistakes, poor reproducibility, and biases in study design. Most studies focused on achieving the best accuracy for their algorithms, sometimes putting aside validity and applicability in clinical practice. Indeed, only two articles presented with an overall low risk of bias.

The common limitations concerned datasets used for the model development, definition of the ground truth for evaluation of the model and strategies used for model evaluation.

Regarding the datasets used, some are private, and some are public open source. For private databases, advanced technical characteristics of images (e.g., imaging sequence, field of view, noise) used and patient’s inclusion and exclusion criteria were poorly or not described. Most databases lacked representability of patients’ variability as prostate volume, prostate tissue heterogeneity, prostatic pathology as PCa or benign hypertropia. Open-source prostate MRI databases also have several limitations such as selection bias, limited annotations, low-resolution images, unclear terminology, lack of demographic statistics and of precise histologic data.

This can have a direct impact on the generalizability of the model developed. Indeed, it has been shown for example that prostate morphological differences contribute to segmentation variability: Montagne et al. [48], showed that the smaller the prostate volume was, the higher the variability was; several authors [18, 39, 43] found poorer performance of their model applied on special cases such as history of trans-urethral-resection of prostate (TURP), while most databases lacked representativity of patients variability.

Even though it is tedious and time-consuming, reference segmentation should require at least two trained readers because inter- and intra-rater variability can be significant. Quality of images (slice thickness, partial volume artifacts), apex or base location [48, 49] or prostate morphological differences [48] have been shown to decrease accuracy of segmentation. Meyer et al. [34] showed that training on segmentation obtained by a single reader introduced bias into the training data. Indeed, performance was higher when obtained from the expert who created the training data in comparison with evaluation against other expert segmentation. Aldoj et al. [39] emphasized the need for finely annotated sets as they improved overall performances of their algorithms, showing the greater importance of well annotated databases compared to large and coarsely annotated databases.

Quality of the resulting auto segmentation is evaluated against the corresponding reference segmentation, so called the ground truth. The main approach is manual delineation of the prostate zones performed by human experts. We found a great heterogeneity on the segmentation protocols and terminology used. Eighteen different types of prostate delineation were found; each anatomical zone was segmented directly or obtained by subtraction from one region to another (resulting in CZ, AFMS and PZ, which can be obtained either by delineation or by subtraction of WG and TZ). Terminology used was extremely variable from one study to another and did not always respect the one used and referenced in the PI-RADS [3, 50] (for example, use of “central gland” instead of CZ or TZ).

Number of readers, level of expertise, inter- and intra-variability evaluation were mostly absent, limiting the generalizability of the developed models due to inter-observer variability. Only 2/33 studies [10, 23] used blinded reading for ground truth. Nonetheless, prostate segmentation is a very challenging task. The prostate gland usually has fuzzy boundaries. Pixel intensities are heterogeneous both inside and outside the prostate, and contrasts and pixel intensities are very similar for prostate and non-prostate regions. The manual delineation of the prostate zones is therefore limited by the subjective interpretation of the organ boundaries. Becker et al. [49] found in a multi-reader study a higher variability at the extreme part of the gland (apex and base) and for the TZ delineation. Similar results were found by Padgett et al. [8] who found a difference of DSC from 0.88 to 0.81 for WG and TZ. Meyer et al. [34] showed that training on segmentation obtained by a single reader introduced bias into the training data.

Strategies used for model evaluation were limited by the lack of external validation Only 7 studies [6, 9, 14, 30, 33, 36, 37] used both private and public data to evaluate their model. The absence of an external testing dataset is a critical limitation to the clinical applicability of the developed models. Data augmentation and transfer learning were also used to help addressing this issue [6, 14–16, 29, 31, 33, 35–41, 43, 51]. It is important to note that some bias cannot be balanced-out by increasing the sample size by data augmentation or repetition of training. For example, data augmentation of a dataset constituted without prostate cancer patients cannot decrease risk of bias induced by the more homogeneous contours it provides.

Even without data augmentation, MRI images contains wide heterogeneity and most of the times pre-processing steps involving intensity normalization or noise reduction to remove confounding features and improve image quality are necessary [52]. Some authors [6, 13, 15, 31, 33, 35, 51] also reported post-processing. Not reporting some of the pre- or post-processing steps can affect reproducibility and sufficient detail enables readers to determine the quality and generalizability of the work. While several checklists can be used such as those from Enhancing the Quality and Transparency Of health Research (EQUATOR) Network guidelines [53], the use of the recently published Checklist for Artificial Intelligence in Medical Imaging [22] would be helpful to lower risk of bias of ongoing work.

In the future, there is a need for well-sampled databases including large number of representative cases for the anatomical variability of the prostate gland and technical specificities (2D T2 versus 3D T2, slice thickness, FOV, vendors) to account for the anatomical, disease related, acquisition related variabilities, with a multi-readers segmentations and a well-defined delineation guideline of the prostate (as it is already done for example in organs at risk for radiotherapy planning [54]).

Constitution of quality database should be based on latest PI-RADS recommendations, by associating quality criteria such as the consensual quality requirements ESUR/ESUI [55] or Prostate Imaging Quality (PI-QUAL) [56] score to guarantee essential image quality for zonal segmentation and tumor detection.

The main limitation of this review is the absence of details of technical information used; each study making its own contribution for networks with countless hyperparameters, sometimes without enough details to be gathered. This precluded us from comparing models’ accuracy without bias.

Some other relevant papers also could be missing because of incongruences between search terms, article keywords, or indexing in the databases, such as for conference proceedings papers. In particular, databases such as ArXiv were not searched as it also provides access to preprints, without peer review.

## Conclusion

This review systematically synthesizes published automatic prostate zonal segmentation methods using MRI. We found that no papers in the literature currently have both sufficiently documented datasets selection and segmentation criteria and enough external validation.

This underlines the critical need for higher quality datasets, a documented reproducible method and terminology for zonal segmentation and sufficient external dataset to develop the best quality methods free from biases: an essential step for future development of automatic detection of prostate cancer.

## Supplementary Information

**Additional file 1: Figure S1.** Schematic of the 17 types of protocol of zonal segmentation. Type A1:Detailed segmentation of WG, PZ and CG (including CZ + TZ + AFMS) [23, 43]. Type A2:Detailed segmentation of WG, CG (including CZ + TZ + AFMS) [25]. Type A3:Detailed segmentation of PZ, CG (including CZ + TZ + AFMS) [36]. Type A4:Segmentation of PZ and urethra only [32]. Type B1a: Segmentation of PZ, CG (including TZ and CZ), without detail for AFMS [31]. Type B1b: Detailed segmentation of PZ, «TZ» (including TZ and CZ), AFMS not segmented [37]. Type B2a: Segmentation of WG, PZ, CG (including TZ + CZ) without detail for AFMS (does not seem segmented) [13]. Type B2b: Segmentation of WG, PZ, «TZ» (including TZ + CZ), without detail for AFMS [8]. Type B2c: Segmentation of WG, PZ, CG (including TZ + CZ) without detail for AFMS (seems segmented with PZ) [9, 26]. Type B3: Detailed segmentation of PZ, CG (including TZ et CZ) and AFMS [34]. Type C1a: Segmentation of PZ, CG without detail for CZ or AFMS [7, 15, 17, 28, 33]. Type C1b: Segmentation of TZ et PZ, with WG = CG + PZ, without detail for CZ or AFMS [10, 16, 29, 41]. Type C2: Segmentation of WG, PZ et CG, without detail for CZ or AFMS [18, 27, 39]. Type C3: Detailed segmentation of WG, «TZ» (including TZ + AFMS). PZ and CZ were not segmented [40]. Type C4:Segmentation of WG and PZ, with WG – PZ = CG, without detail for CZ or AFMS [6, 12]. Type D1a:Segmentation of WG, TZ without detail for CZ or AFMS [14, 35, 38]. Type D1b:Segmentation of WG, CG, without detail for CZ or AFMS [24, 30]. Type D2:Segmentation of WG, TZ, PZ, without detail for CZ or AFMS [42]. Segmentation protocols details not reported in full text were extrapolated from figures of those articles. CZ: central zone. TZ: transition zone. AFMS: anterior fibro-muscular stroma. PZ: peripheral zone. CG: central gland. WG: whole gland. **Table S1.** Overview of most used public databases characteristics. **Table S2. **Detailed quality assessment for risk of bias and applicability of included studies.

## Data Availability

All data generated or analyzed during this study are included in this published article [and its supplementary information files].

## Abbreviations

AFMS: Anterior fibro-muscular stroma
CG: Central gland
CZ: Central zone
DSC: Dice similarity coefficient
FOV: Field of view
PCa: Prostate cancer
PZ: Peripheral zone
QUADAS-2: Quality Assessment of Diagnostic Accuracy Studies 2
TZ: Transition zone
WG: Whole gland
