# Supplementary material for: Automatic segmentation of prostate zonal anatomy on MRI: a systematic review of the literature
Source: Insights Imaging. 2022 Dec 21;13:202. doi: 10.1186/s13244-022-01340-2 (PMC9772373; doi:10.1186/s13244-022-01340-2)
Supplement: Supplementary file 1 — Additional file 1: Figure S1. Schematic of the 17 types of protocol of zonal segmentation. Type A1:Detailed segmentation of WG, PZ and CG (including CZ + TZ + AFMS) [23, 43]. Type A2:Detailed segmentation of WG, CG (including CZ + TZ + AFMS) [25]. Type A3:Detailed segmentation of PZ, CG (including CZ + TZ + AFMS) [36]. Type A4:Segmentation of PZ and urethra only [32]. Type B1a: Segmentation of PZ, CG (including TZ and CZ), without detail for AFMS [31]. Type B1b: Detailed segmentation of PZ, «TZ» (including TZ and CZ), AFMS not segmented [37]. Type B2a: Segmentation of WG, PZ, CG (including TZ + CZ) without detail for AFMS (does not seem segmented) [13]. Type B2b: Segmentation of WG, PZ, «TZ» (including TZ + CZ), without detail for AFMS [8]. Type B2c: Segmentation of WG, PZ, CG (including TZ + CZ) without detail for AFMS (seems segmented with PZ) [9, 26]. Type B3: Detailed segmentation of PZ, CG (including TZ et CZ) and AFMS [34]. Type C1a: Segmentation of PZ, CG without detail for CZ or AFMS [7, 15, 17, 28, 33]. Type C1b: Segmentation of TZ et PZ, with WG = CG + PZ, without detail for CZ or AFMS [10, 16, 29, 41]. Type C2: Segmentation of WG, PZ et CG, without detail for CZ or AFMS [18, 27, 39]. Type C3: Detailed segmentation of WG, «TZ» (including TZ + AFMS). PZ and CZ were not segmented [40]. Type C4:Segmentation of WG and PZ, with WG – PZ = CG, without detail for CZ or AFMS [6, 12]. Type D1a:Segmentation of WG, TZ without detail for CZ or AFMS [14, 35, 38]. Type D1b:Segmentation of WG, CG, without detail for CZ or AFMS [24, 30]. Type D2:Segmentation of WG, TZ, PZ, without detail for CZ or AFMS [42]. Segmentation protocols details not reported in full text were extrapolated from figures of those articles. CZ: central zone. TZ: transition zone. AFMS: anterior fibro-muscular stroma. PZ: peripheral zone. CG: central gland. WG: whole gland. Table S1. Overview of most used public databases characteristics. Table S2. Detailed quality assessment for risk of bias and appli [file 13244_2022_1340_MOESM1_ESM.pdf]

ELECTRONIC SUPPLEMENTARY MATERIAL

Automatic segmentation of prostate zonal anatomy on MRI: a systematic review of the literature

Supplementary table 1: Overview of most used public databases characteristics

	PROMISE12 [45]	NCI-ISBI [44]	PROSTATEx [43]
**Inclusion criteria**	✓	✗	✓
**Number of patients**	100 (train) + 50 (live)	60	346
**Field strength**	1.5T and 3T	1.5T and 3T	3T
**Coil Type**	ERC and SC	ERC and SC	SC
**Axial T2W slice thickness**	3 – 3.6mm on 1.5T<br>2.2 – 4 mm on 3T	3 mm on 1.5T<br>4 mm on 3T	3.6 mm
**Number of vendors**	2	2	1
**Multicentric**	✓	✓	✗
**Segmentation WG**	✓	✓	✗
**Zonal Segmentation**	✗	✓	✗
**Presence of PCa**	✓	?	✓

ERC: endorectal coil

SC: surface coil

WG: whole gland

PCa: prostate cancer lesion

?: not reported

Insights Imaging (2022) Wu C, Montagne S, Hamzaoui D, Ayache N, Delingette H, Rennard Penna R

Supplementary table 2: Detailed quality assessment for risk of bias and applicability of included studies.

	Risk of bias				Applicability		
**First author, year of publication**	**Patient selection**	**Index Test**	**Reference standard**	**Flow and timing**	**Patient selection**	**Index Test**	**Reference Standard**
**Lai, 2021**	😐	✅	❌	✅	😐	✅	😐
**Bardis, 2021**	✅	✅	😐	✅	✅	✅	😐
**Cuocolo, 2021**	😐	✅	✅	✅	😐	✅	✅
**Qin, 2020**	😐	✅	❌	❌	✅	✅	✅
**Lee, 2020**	✅	✅	😐	✅	😐	✅	😐
**Motamed, 2020**	😐	✅	😐	✅	😐	✅	😐
**Sanford, 2020**	✅	✅	❌	✅	✅	✅	✅
**Aldoj, 2020**	😐	✅	❌	✅	😐	✅	❌
**Liu, 2020**	✅	✅	✅	✅	😐	✅	😐
**Nai,2020**	😐	✅	✅	✅	😐	✅	✅
**Zavala-Romero, 2020**	✅	✅	✅	❌	✅	✅	✅
**Rundo[1], 2019**	❌	✅	😐	😐	😐	✅	✅
**Rundo[2], 2019**	😐	✅	😐	😐	😐	✅	✅

Insights Imaging (2022) Wu C, Montagne S, Hamzaoui D, Ayache N, Delingette H, Rennard Penna R

**Hambarde, 2019**	✗	✓	😐	✓	😐	✓	✓
**Khan, 2019**	✗	✓	😐	✓	😐	✓	😐
**Zhu, 2019**	✓	✓	😐	✓	😐	✓	✓
**Jensen, 2019**	😐	✓	✗	✓	✓	😐	✓
**Liu, 2019**	✓	✓	✓	✓	😐	✓	😐
**Meyer, 2019**	😐	✓	✓	✓	😐	✓	✓
**Zabihohally, 2019**	✓	✓	😐	✓	😐	✓	✓
**Padgett, 2019**	😐	✓	✓	😐	😐	✓	✓
**Cheng, 2019**	😐	✓	✗	✗	😐	✓	😐
**Mooij, 2018**	✗	✓	✗	✓	✗	✓	😐
**Can, 2018**	✗	✓	😐	✓	😐	✓	😐
**Clark, 2017**	😐	✓	✗	✗	😐	✓	😐
**Chilali, 2016**	😐	✓	✗	✓	✓	✓	✓
**Chi, 2014**	✗	✓	✗	✓	✗	😐	✗
**Makni, 2014**	😐	✓	✓	✓	😐	✓	✓
**Toth, 2013**	😐	✓	✗	✗	😐	✓	✓
**Litjens, 2012**	✗	✓	✓	✗	✗	😐	✓
**Moschidis, 2012**	✗	✓	😐	✗	😐	✓	✓

Insights Imaging (2022) Wu C, Montagne S, Hamzaoui D, Ayache N, Delingette H, Rennard Penna R

**Yin, 2012**	❌	✅	❌	✅	❌	😐	😐
**Makni, 2011**	😐	✅	✅	😐	😐	😐	✅

1: Rundo et al., USE-Net: incorporating Squeeze-and-Excitation blocks into U-Net for prostate zonal segmentation of multi-institutional MRI datasets [30]

2: Rundo et al., CNN-based Prostate Zonal Segmentation on T2-weighted MR Images: A Cross-dataset Study [27]

Supplementary Figure 1

Supplementary Figure 1: Schematic of the 17 types of protocol of zonal segmentation
Type A1:**Detailed segmentation** of WG, PZ and CG (including CZ + TZ + AFMS) [23, 43]
Type A2:**Detailed segmentation** of WG, CG (including CZ + TZ + AFMS) [25]
Type A3:**Detailed segmentation** of PZ, CG (including CZ + TZ + AFMS) [36]
Type A4:Segmentation of PZ and urethra only [32]

Type B1a: Segmentation of PZ, CG (including TZ and CZ), without detail for AFMS [31]
Type B1b: **Detailed segmentation** of PZ, « TZ » (including TZ and CZ), AFMS not segmented [37]
Type B2a: Segmentation of WG, PZ, CG (including TZ + CZ) without detail for AFMS (does not seem segmented) [13]
Type B2b: Segmentation of WG, PZ, « TZ » (including TZ + CZ), without detail for AFMS [8]
Type B2c: Segmentation of WG, PZ, CG (including TZ + CZ) without detail for AFMS (seems segmented with PZ) [9, 26]
Type B3: **Detailed segmentation** of PZ, CG (including TZ et CZ) and AFMS [34]

Type C1a: Segmentation of PZ, CG without detail for CZ or AFMS [7, 15, 17, 28, 33]
Type C1b: Segmentation of TZ et PZ, with WG = CG + PZ, without detail for CZ or AFMS [10, 16, 29, 41]
Type C2: Segmentation of WG, PZ et CG, without detail for CZ or AFMS [18, 27, 39]
Type C3: **Detailed segmentation** of WG, « TZ » (including TZ + AFMS). PZ and CZ were not segmented [40]
Type C4:Segmentation of WG and PZ, with WG – PZ = CG, without detail for CZ or AFMS [6, 12]

Type D1a:Segmentation of WG, TZ without detail for CZ or AFMS [14, 35, 38]
Type D1b:Segmentation of WG, CG, without detail for CZ or AFMS [24, 30]
Type D2:Segmentation of WG, TZ, PZ, without detail for CZ or AFMS [42]

Segmentation protocols details not reported in full text were extrapolated from figures of those articles

CZ:central zone
TZ:transition zone
AFMS:anterior fibro muscular stroma
PZ:peripheral zone
CG:central gland
WG:whole gland

A:

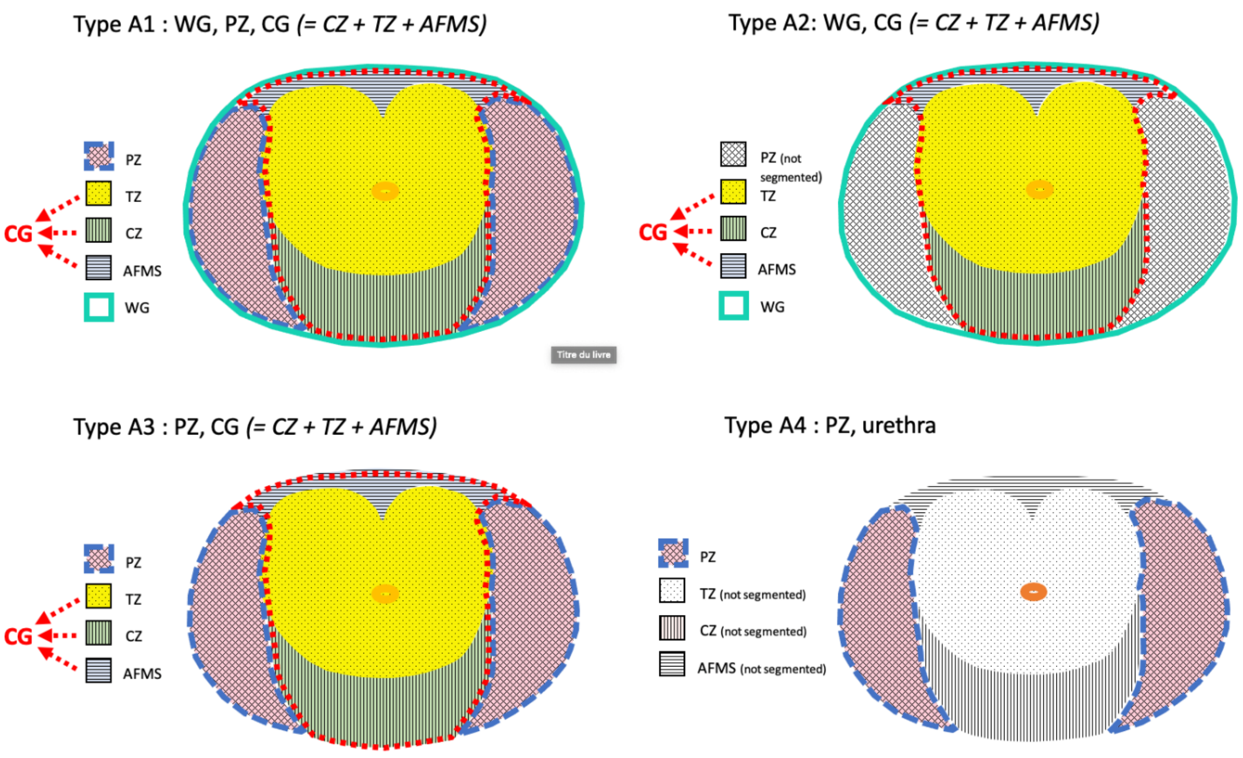

Insights Imaging (2022) Wu C, Montagne S, Hamzaoui D, Ayache N, Delingette H, Rennard Penna R

B:

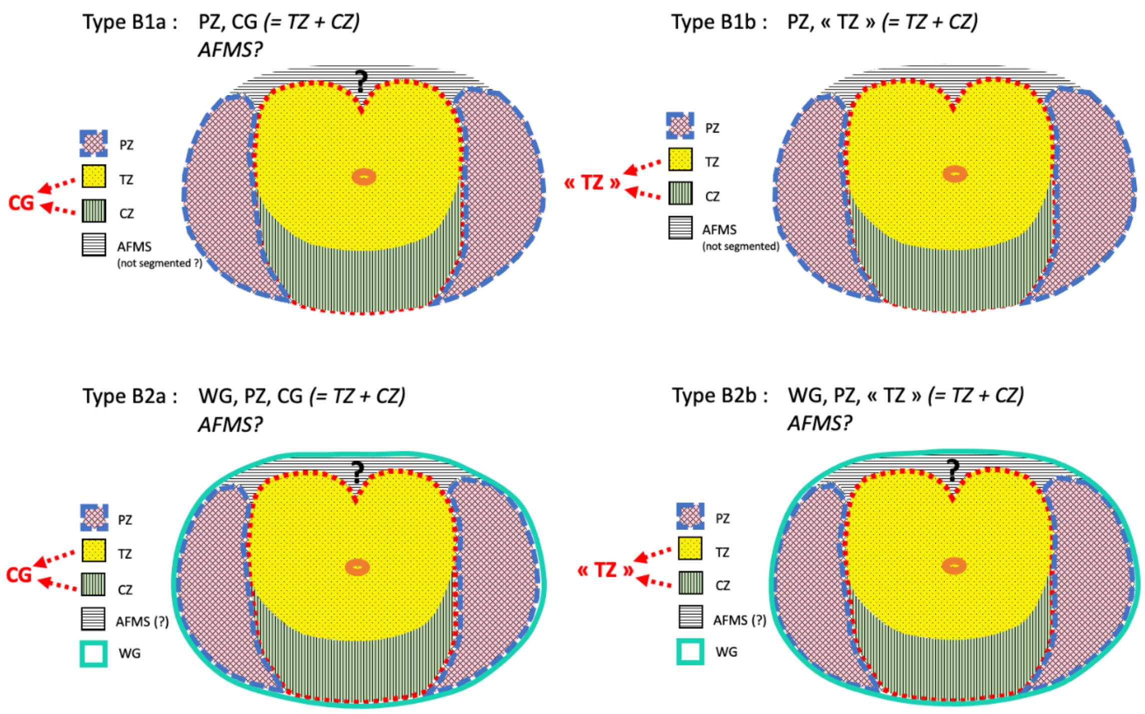
Type B1a : PZ, CG (= TZ + CZ)
AFMS?
?
PZ
TZ
CZ
AFMS
(not segmented ?)
CG
Type B1b : PZ, « TZ » (= TZ + CZ)
PZ
TZ
CZ
AFMS
(not segmented)
« TZ »
Type B2a : WG, PZ, CG (= TZ + CZ)
AFMS?
?
PZ
TZ
CZ
AFMS (?)
WG
CG
Type B2b : WG, PZ, « TZ » (= TZ + CZ)
AFMS?
?
PZ
TZ
CZ
AFMS (?)
WG
« TZ »

C:

Type B2c : WG, PZ, CG *(= TZ + CZ)*
AFMS?

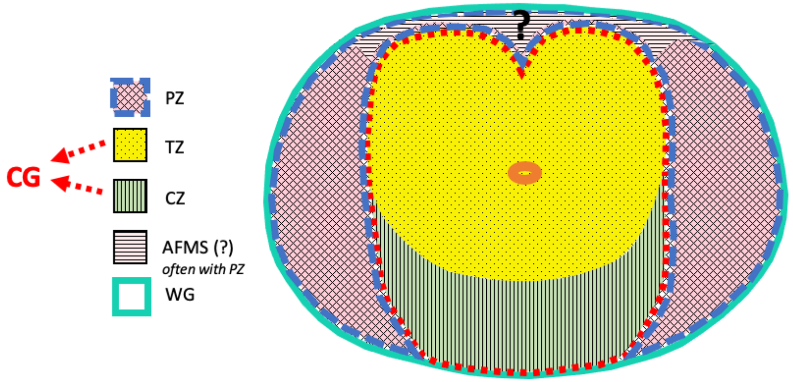

Type B3 : PZ, CG *(= TZ + CZ)*, AFMS

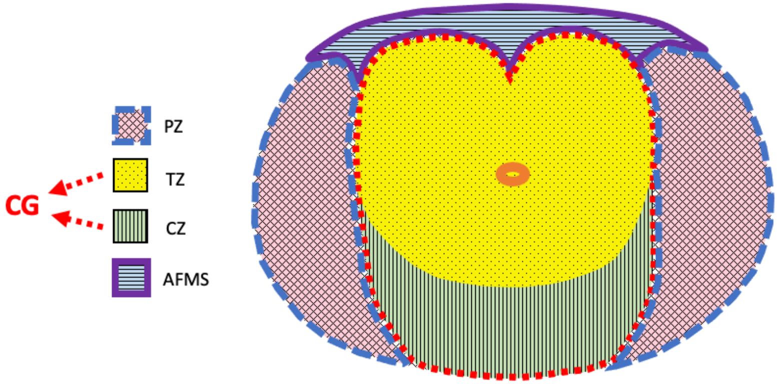

Insights Imaging (2022) Wu C, Montagne S, Hamzaoui D, Ayache N, Delingette H, Rennard Penna R

D:

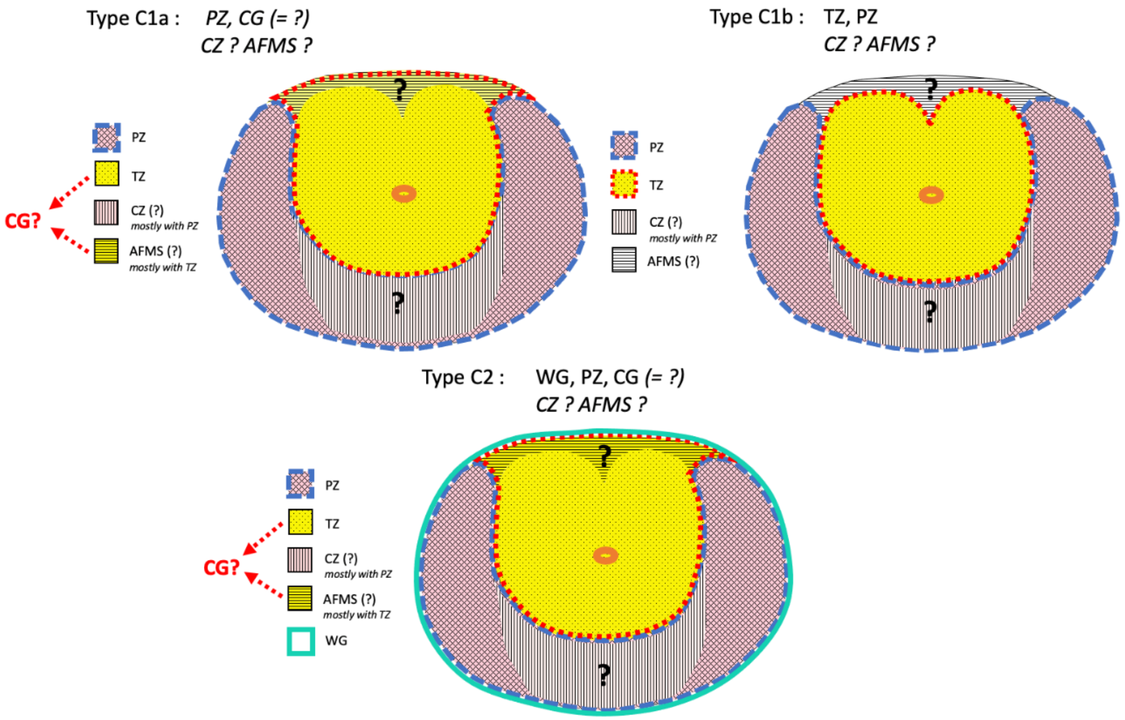

Insights Imaging (2022) Wu C, Montagne S, Hamzaoui D, Ayache N, Delingette H, Rennard Penna R

E:

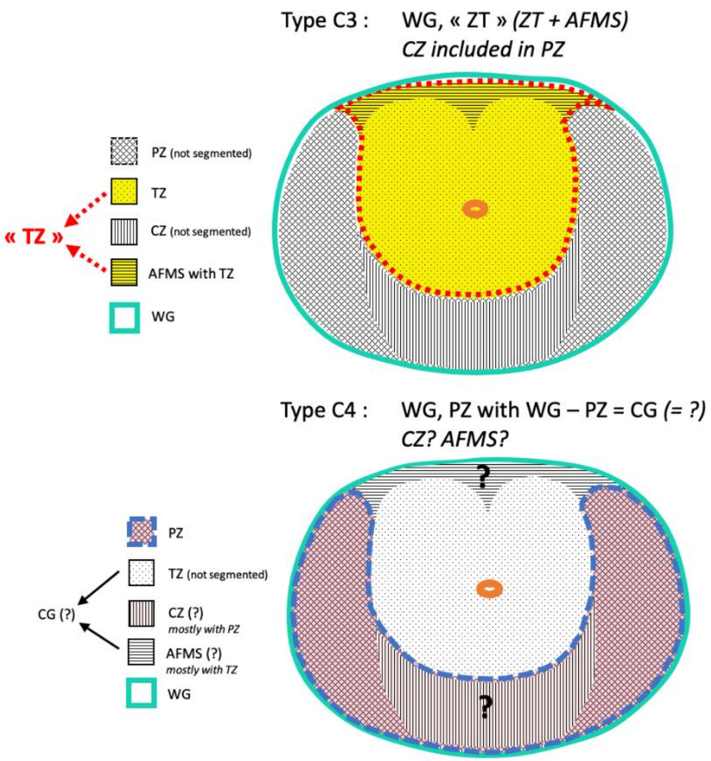

Type C3 :
WG, « ZT » (ZT + AFMS)
CZ included in PZ
PZ (not segmented)
TZ
CZ (not segmented)
AFMS with TZ
WG
« TZ »
Type C4 :
WG, PZ with WG – PZ = CG (= ?)
CZ? AFMS?
?
?
PZ
TZ (not segmented)
CZ (?)
mostly with PZ
AFMS (?)
mostly with TZ
WG
CG (?)

F:

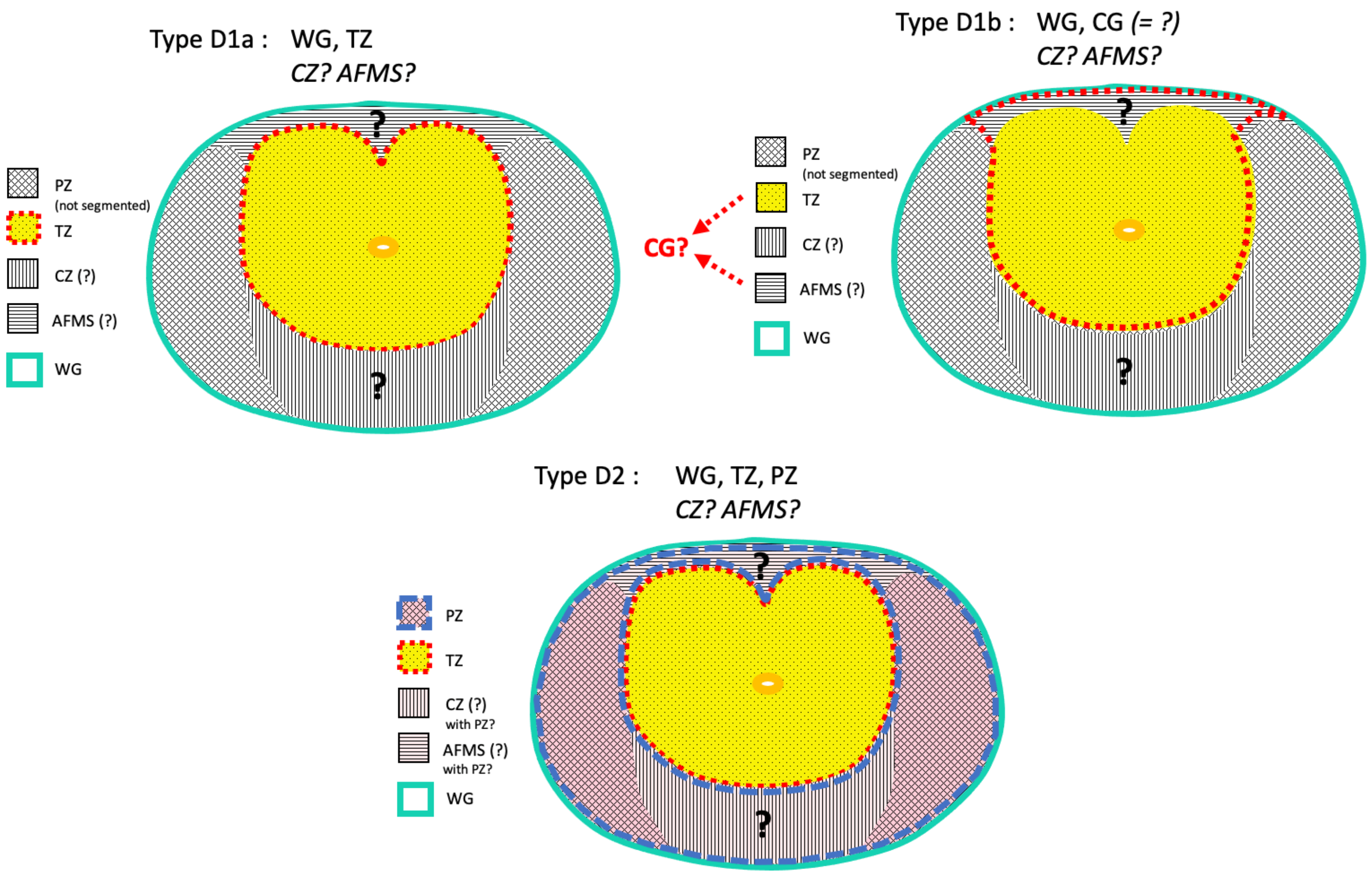

Insights Imaging (2022) Wu C, Montagne S, Hamzaoui D, Ayache N, Delingette H, Rennard Penna R
